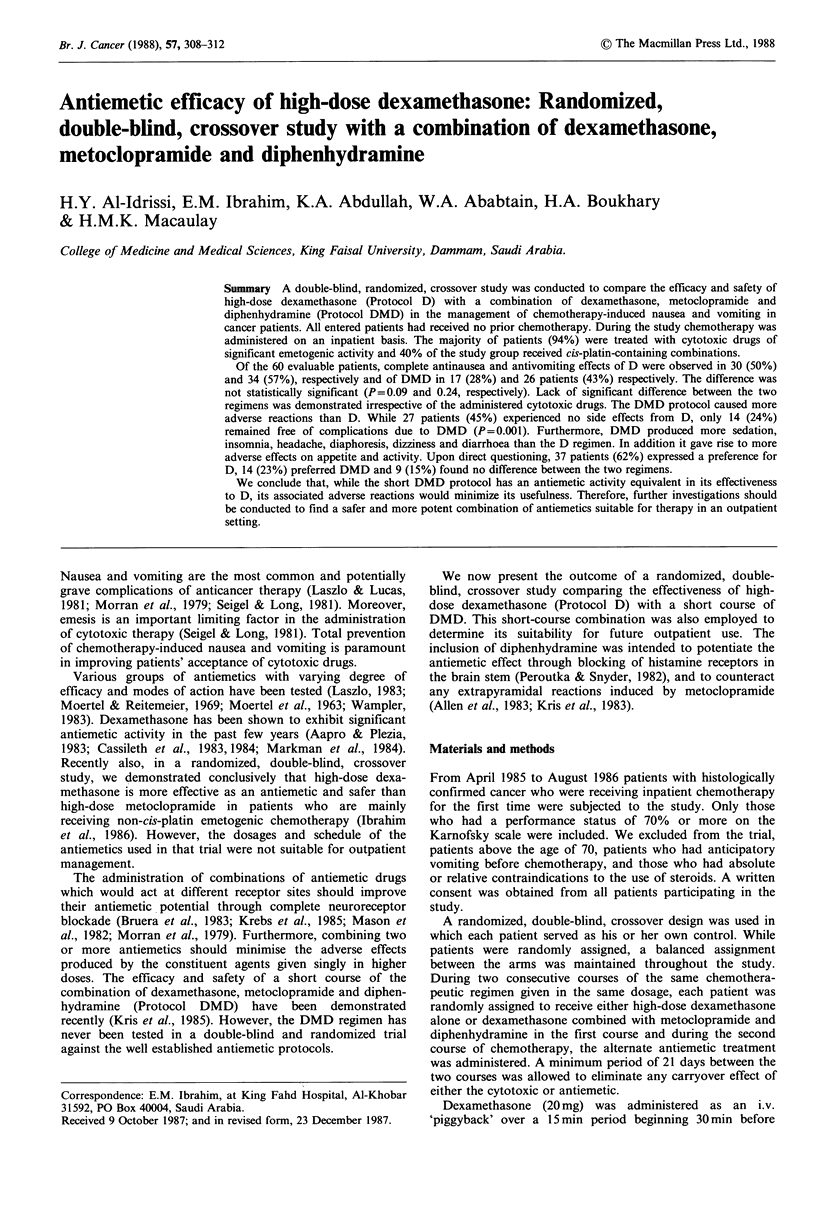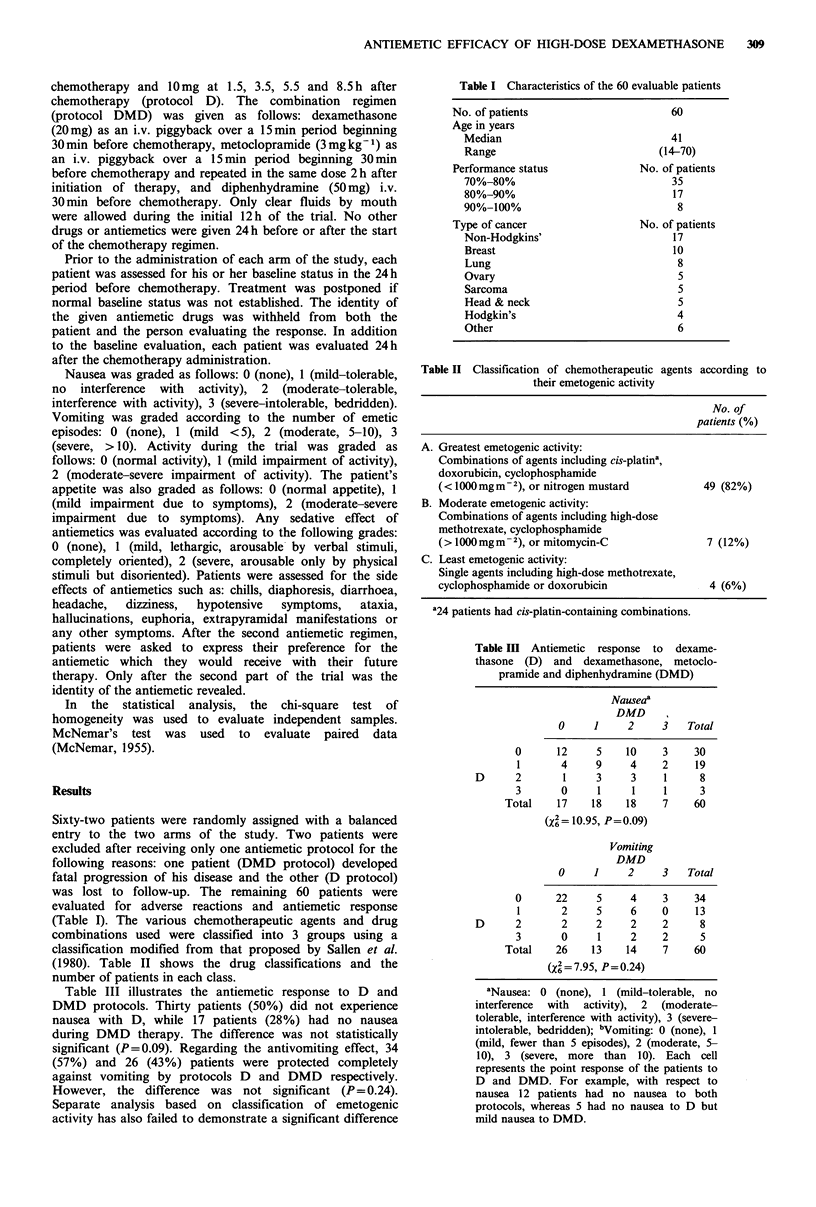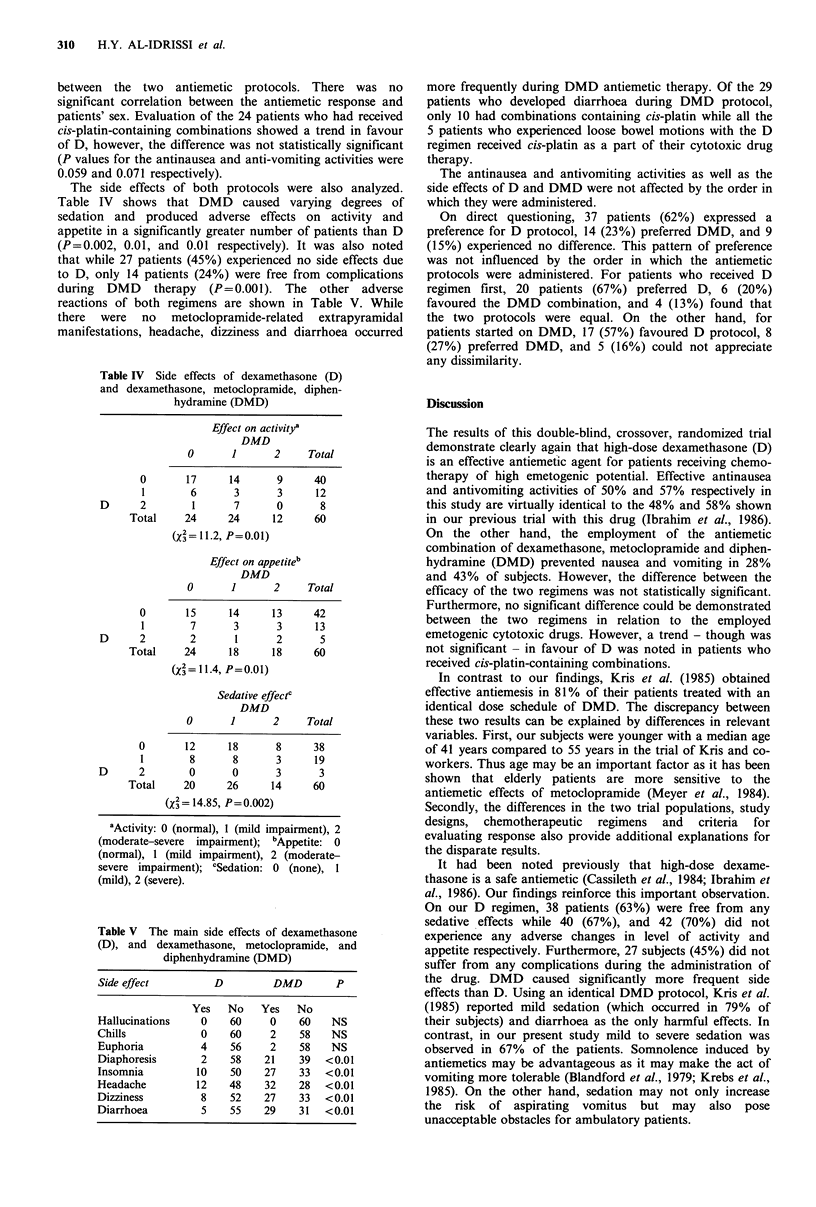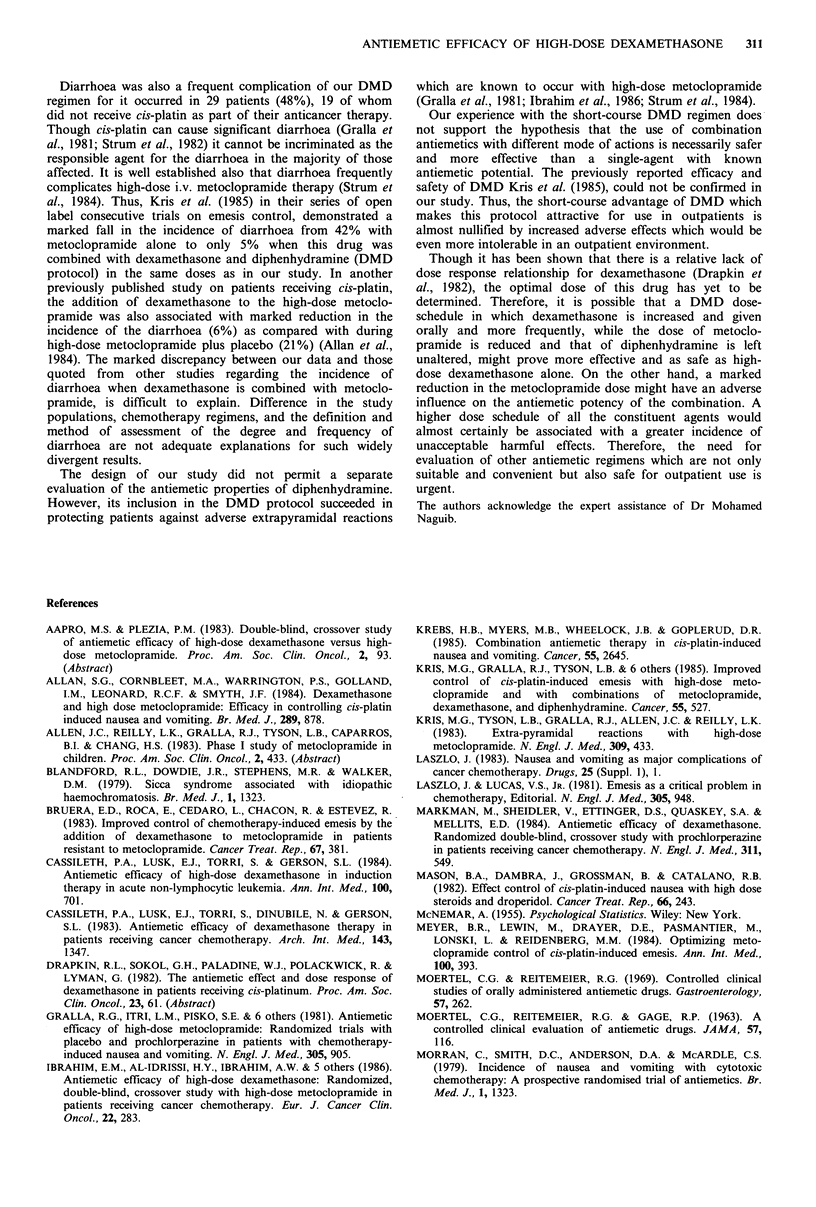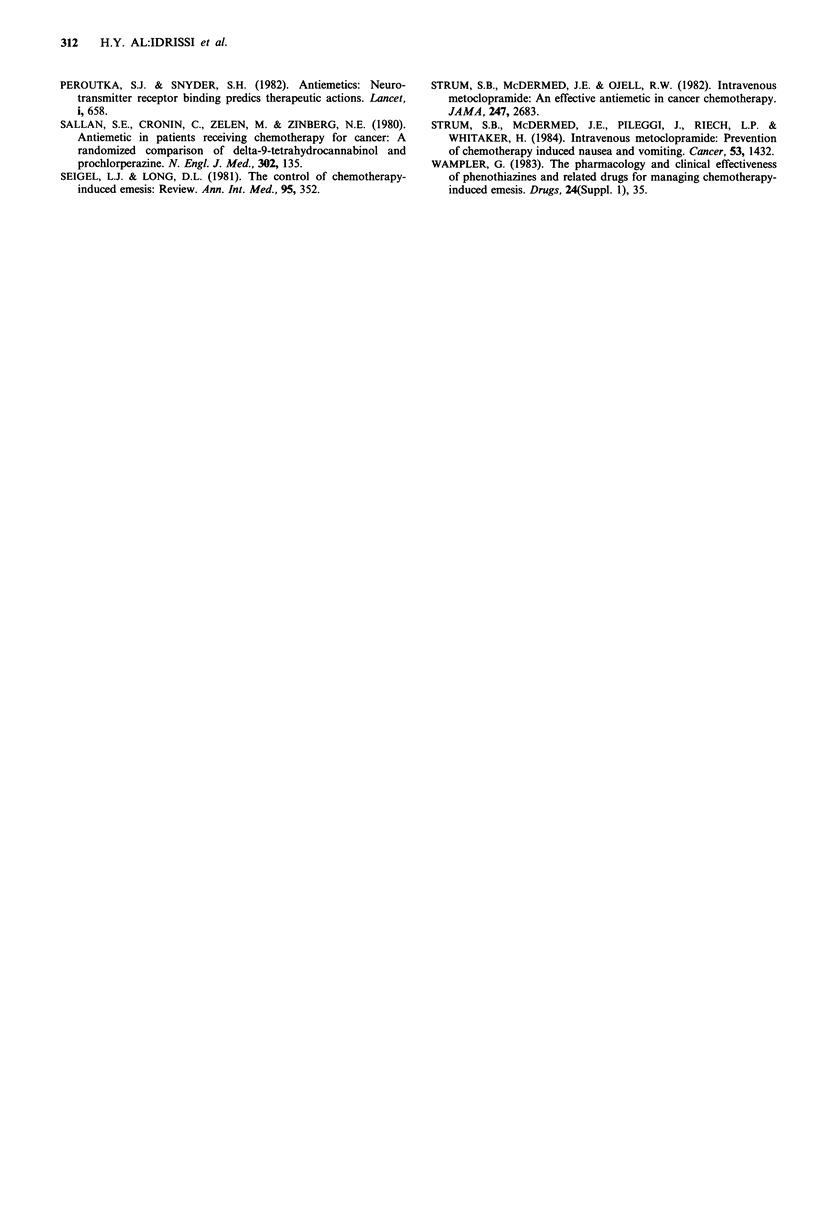# Antiemetic efficacy of high-dose dexamethasone: randomized, double-blind, crossover study with a combination of dexamethasone, metoclopramide and diphenhydramine.

**DOI:** 10.1038/bjc.1988.68

**Published:** 1988-03

**Authors:** H. Y. al-Idrissi, E. M. Ibrahim, K. A. Abdullah, W. A. Ababtain, H. A. Boukhary, H. M. Macaulay

**Affiliations:** College of Medicine and Medical Sciences, King Faisal University, Dammam, Saudi Arabia.

## Abstract

A double-blind, randomized, crossover study was conducted to compare the efficacy and safety of high-dose dexamethasone (Protocol D) with a combination of dexamethasone, metoclopramide and diphenhydramine (Protocol DMD) in the management of chemotherapy-induced nausea and vomiting in cancer patients. All entered patients had received no prior chemotherapy. During the study chemotherapy was administered on an inpatient basis. The majority of patients (94%) were treated with cytotoxic drugs of significant emetogenic activity and 40% of the study group received cis-platin-containing combinations. Of the 60 evaluable patients, complete antinausea and antivomiting effects of D were observed in 30 (50%) and 34 (57%), respectively and of DMD in 17 (28%) and 26 patients (43%) respectively. The difference was not statistically significant (P = 0.09 and 0.24, respectively). Lack of significant difference between the two regimens was demonstrated irrespective of the administered cytotoxic drugs. The DMD protocol caused more adverse reactions than D. While 27 patients (45%) experienced no side effects from D, only 14 (24%) remained free of complications due to DMD (P = 0.001). Furthermore, DMD produced more sedation, insomnia, headache, diaphoresis, dizziness and diarrhoea than the D regimen. In addition it gave rise to more adverse effects on appetite and activity. Upon direct questioning, 37 patients (62%) expressed a preference for D, 14 (23%) preferred DMD and 9 (15%) found no difference between the two regimens. We conclude that, while the short DMD protocol has an antiemetic activity equivalent in its effectiveness to D, its associated adverse reactions would minimize its usefulness. Therefore, further investigations should be conducted to find a safer and more potent combination of antiemetics suitable for therapy in an outpatient setting.


Br. J. Cancer (1988), 57, 308–312 © The Macmillan Press Ltd., 1988

### Antiemetic efficacy of high-dose dexamethasone: Randomized, double-blind, crossover study with a combination of dexamethasone, metoclopramide and diphenhydramine

H.Y. Al-Idrissi, E.M. Ibrahim, K.A. Abdullah, W.A. Ababtain, H.A. Boukhary & H.M.K. Macaulay

*College of Medicine and Medical Sciences, King Faisal University, Dammam, Saudi Arabia.*

**Summary** A double-blind, randomized, crossover study was conducted to compare the efficacy and safety of high-dose dexamethasone (Protocol D) with a combination of dexamethasone, metoclopramide and diphenhydramine (Protocol DMD) in the management of chemotherapy-induced nausea and vomiting in cancer patients. All entered patients had received no prior chemotherapy. During the study chemotherapy was administered on an inpatient basis. The majority of patients (94%) were treated with cytotoxic drugs of significant emetogenic activity and 40% of the study group received *cis*-platin-containing combinations.

Of the 60 evaluable patients, complete antinausea and antivomiting effects of D were observed in 30 (50%) and 34 (57%), respectively and of DMD in 17 (28%) and 26 patients (43%) respectively. The difference was not statistically significant ($P = 0.09$ and 0.24, respectively). Lack of significant difference between the two regimens was demonstrated irrespective of the administered cytotoxic drugs. The DMD protocol caused more adverse reactions than D. While 27 patients (45%) experienced no side effects from D, only 14 (24%) remained free of complications due to DMD ($P = 0.001$). Furthermore, DMD produced more sedation, insomnia, headache, diaphoresis, dizziness and diarrhoea than the D regimen. In addition it gave rise to more adverse effects on appetite and activity. Upon direct questioning, 37 patients (62%) expressed a preference for D, 14 (23%) preferred DMD and 9 (15%) found no difference between the two regimens.

We conclude that, while the short DMD protocol has an antiemetic activity equivalent in its effectiveness to D, its associated adverse reactions would minimize its usefulness. Therefore, further investigations should be conducted to find a safer and more potent combination of antiemetics suitable for therapy in an outpatient setting.

Nausea and vomiting are the most common and potentially grave complications of anticancer therapy (Laszlo & Lucas, 1981; Morran *et al.*, 1979; Seigel & Long, 1981). Moreover, emesis is an important limiting factor in the administration of cytotoxic therapy (Seigel & Long, 1981). Total prevention of chemotherapy-induced nausea and vomiting is paramount in improving patients' acceptance of cytotoxic drugs.

Various groups of antiemetics with varying degree of efficacy and modes of action have been tested (Laszlo, 1983; Moertel & Reitemeier, 1969; Moertel *et al.*, 1963; Wampler, 1983). Dexamethasone has been shown to exhibit significant antiemetic activity in the past few years (Aapro & Plezia, 1983; Cassileth *et al.*, 1983, 1984; Markman *et al.*, 1984). Recently also, in a randomized, double-blind, crossover study, we demonstrated conclusively that high-dose dexamethasone is more effective as an antiemetic and safer than high-dose metoclopramide in patients who are mainly receiving non-*cis*-platin emetogenic chemotherapy (Ibrahim *et al.*, 1986). However, the dosages and schedule of the antiemetics used in that trial were not suitable for outpatient management.

The administration of combinations of antiemetic drugs which would act at different receptor sites should improve their antiemetic potential through complete neuroreceptor blockade (Bruera *et al.*, 1983; Krebs *et al.*, 1985; Mason *et al.*, 1982; Morran *et al.*, 1979). Furthermore, combining two or more antiemetics should minimise the adverse effects produced by the constituent agents given singly in higher doses. The efficacy and safety of a short course of the combination of dexamethasone, metoclopramide and diphenhydramine (Protocol DMD) have been demonstrated recently (Kris *et al.*, 1985). However, the DMD regimen has never been tested in a double-blind and randomized trial against the well established antiemetic protocols.

Correspondence: E.M. Ibrahim, at King Fahd Hospital, Al-Khobar 31592, PO Box 40004, Saudi Arabia.
Received 9 October 1987; and in revised form, 23 December 1987.

We now present the outcome of a randomized, double-blind, crossover study comparing the effectiveness of high-dose dexamethasone (Protocol D) with a short course of DMD. This short-course combination was also employed to determine its suitability for future outpatient use. The inclusion of diphenhydramine was intended to potentiate the antiemetic effect through blocking of histamine receptors in the brain stem (Peroutka & Snyder, 1982), and to counteract any extrapyramidal reactions induced by metoclopramide (Allen *et al.*, 1983; Kris *et al.*, 1983).

### Materials and methods

From April 1985 to August 1986 patients with histologically confirmed cancer who were receiving inpatient chemotherapy for the first time were subjected to the study. Only those who had a performance status of 70% or more on the Karnofsky scale were included. We excluded from the trial, patients above the age of 70, patients who had anticipatory vomiting before chemotherapy, and those who had absolute or relative contraindications to the use of steroids. A written consent was obtained from all patients participating in the study.

A randomized, double-blind, crossover design was used in which each patient served as his or her own control. While patients were randomly assigned, a balanced assignment between the arms was maintained throughout the study. During two consecutive courses of the same chemotherapeutic regimen given in the same dosage, each patient was randomly assigned to receive either high-dose dexamethasone alone or dexamethasone combined with metoclopramide and diphenhydramine in the first course and during the second course of chemotherapy, the alternate antiemetic treatment was administered. A minimum period of 21 days between the two courses was allowed to eliminate any carryover effect of either the cytotoxic or antiemetic.

Dexamethasone (20 mg) was administered as an i.v. 'piggyback' over a 15 min period beginning 30 min before chemotherapy and 10 mg at 1.5, 3.5, 5.5 and 8.5 h after chemotherapy (protocol D). The combination regimen (protocol DMD) was given as follows: dexamethasone (20 mg) as an i.v. piggyback over a 15 min period beginning 30 min before chemotherapy, metoclopramide (3 mg $kg^{-1}$) as an i.v. piggyback over a 15 min period beginning 30 min before chemotherapy and repeated in the same dose 2 h after initiation of therapy, and diphenhydramine (50 mg) i.v. 30 min before chemotherapy. Only clear fluids by mouth were allowed during the initial 12 h of the trial. No other drugs or antiemetics were given 24 h before or after the start of the chemotherapy regimen.

Prior to the administration of each arm of the study, each patient was assessed for his or her baseline status in the 24 h period before chemotherapy. Treatment was postponed if normal baseline status was not established. The identity of the given antiemetic drugs was withheld from both the patient and the person evaluating the response. In addition to the baseline evaluation, each patient was evaluated 24 h after the chemotherapy administration.

Nausea was graded as follows: 0 (none), 1 (mild–tolerable, no interference with activity), 2 (moderate–tolerable, interference with activity), 3 (severe–intolerable, bedridden). Vomiting was graded according to the number of emetic episodes: 0 (none), 1 (mild $<5$), 2 (moderate, 5–10), 3 (severe, $>10$). Activity during the trial was graded as follows: 0 (normal activity), 1 (mild impairment of activity), 2 (moderate–severe impairment of activity). The patient's appetite was also graded as follows: 0 (normal appetite), 1 (mild impairment due to symptoms), 2 (moderate–severe impairment due to symptoms). Any sedative effect of antiemetics was evaluated according to the following grades: 0 (none), 1 (mild, lethargic, arousable by verbal stimuli, completely oriented), 2 (severe, arousable only by physical stimuli but disoriented). Patients were assessed for the side effects of antiemetics such as: chills, diaphoresis, diarrhoea, headache, dizziness, hypotensive symptoms, ataxia, hallucinations, euphoria, extrapyramidal manifestations or any other symptoms. After the second antiemetic regimen, patients were asked to express their preference for the antiemetic which they would receive with their future therapy. Only after the second part of the trial was the identity of the antiemetic revealed.

In the statistical analysis, the chi-square test of homogeneity was used to evaluate independent samples. McNemar's test was used to evaluate paired data (McNemar, 1955).

### Results

Sixty-two patients were randomly assigned with a balanced entry to the two arms of the study. Two patients were excluded after receiving only one antiemetic protocol for the following reasons: one patient (DMD protocol) developed fatal progression of his disease and the other (D protocol) was lost to follow-up. The remaining 60 patients were evaluated for adverse reactions and antiemetic response (Table I). The various chemotherapeutic agents and drug combinations used were classified into 3 groups using a classification modified from that proposed by Sallen *et al.* (1980). Table II shows the drug classifications and the number of patients in each class.

Table III illustrates the antiemetic response to D and DMD protocols. Thirty patients (50%) did not experience nausea with D, while 17 patients (28%) had no nausea during DMD therapy. The difference was not statistically significant ($P=0.09$). Regarding the antivomiting effect, 34 (57%) and 26 (43%) patients were protected completely against vomiting by protocols D and DMD respectively. However, the difference was not significant ($P=0.24$). Separate analysis based on classification of emetogenic activity has also failed to demonstrate a significant difference

**Table I** Characteristics of the 60 evaluable patients

| | |
|---|---|
| No. of patients | 60 |
| Age in years | |
| Median | 41 |
| Range | (14–70) |
| Performance status | No. of patients |
| 70%–80% | 35 |
| 80%–90% | 17 |
| 90%–100% | 8 |
| Type of cancer | No. of patients |
| Non-Hodgkins' | 17 |
| Breast | 10 |
| Lung | 8 |
| Ovary | 5 |
| Sarcoma | 5 |
| Head & neck | 5 |
| Hodgkin's | 4 |
| Other | 6 |

**Table II** Classification of chemotherapeutic agents according to their emetogenic activity

| | *No. of patients* (%) |
|---|---|
| A. Greatest emetogenic activity: Combinations of agents including *cis*-platin[a], doxorubicin, cyclophosphamide (<1000 mg $m^{-2}$), or nitrogen mustard | 49 (82%) |
| B. Moderate emetogenic activity: Combinations of agents including high-dose methotrexate, cyclophosphamide (>1000 mg $m^{-2}$), or mitomycin-C | 7 (12%) |
| C. Least emetogenic activity: Single agents including high-dose methotrexate, cyclophosphamide or doxorubicin | 4 (6%) |

[a]24 patients had *cis*-platin-containing combinations.

**Table III** Antiemetic response to dexamethasone (D) and dexamethasone, metoclopramide and diphenhydramine (DMD)

| | | *Nausea*[a] DMD 0 | 1 | 2 | 3 | *Total* |
|---|---|---|---|---|---|---|
| | 0 | 12 | 5 | 10 | 3 | 30 |
| | 1 | 4 | 9 | 4 | 2 | 19 |
| D | 2 | 1 | 3 | 3 | 1 | 8 |
| | 3 | 0 | 1 | 1 | 1 | 3 |
| | Total | 17 | 18 | 18 | 7 | 60 |

($\chi_6^2=10.95$, $P=0.09$)

| | | *Vomiting* DMD 0 | 1 | 2 | 3 | *Total* |
|---|---|---|---|---|---|---|
| | 0 | 22 | 5 | 4 | 3 | 34 |
| | 1 | 2 | 5 | 6 | 0 | 13 |
| D | 2 | 2 | 2 | 2 | 2 | 8 |
| | 3 | 0 | 1 | 2 | 2 | 5 |
| | Total | 26 | 13 | 14 | 7 | 60 |

($\chi_6^2=7.95$, $P=0.24$)

[a]Nausea: 0 (none), 1 (mild–tolerable, no interference with activity), 2 (moderate–tolerable, interference with activity), 3 (severe–intolerable, bedridden); [b]Vomiting: 0 (none), 1 (mild, fewer than 5 episodes), 2 (moderate, 5–10), 3 (severe, more than 10). Each cell represents the point response of the patients to D and DMD. For example, with respect to nausea 12 patients had no nausea to both protocols, whereas 5 had no nausea to D but mild nausea to DMD.

between the two antiemetic protocols. There was no significant correlation between the antiemetic response and patients' sex. Evaluation of the 24 patients who had received *cis*-platin-containing combinations showed a trend in favour of D, however, the difference was not statistically significant (*P* values for the antinausea and anti-vomiting activities were 0.059 and 0.071 respectively).

The side effects of both protocols were also analyzed. Table IV shows that DMD caused varying degrees of sedation and produced adverse effects on activity and appetite in a significantly greater number of patients than D ($P=0.002$, 0.01, and 0.01 respectively). It was also noted that while 27 patients (45%) experienced no side effects due to D, only 14 patients (24%) were free from complications during DMD therapy ($P=0.001$). The other adverse reactions of both regimens are shown in Table V. While there were no metoclopramide-related extrapyramidal manifestations, headache, dizziness and diarrhoea occurred more frequently during DMD antiemetic therapy. Of the 29 patients who developed diarrhoea during DMD protocol, only 10 had combinations containing *cis*-platin while all the 5 patients who experienced loose bowel motions with the D regimen received *cis*-platin as a part of their cytotoxic drug therapy.

The antinausea and antivomiting activities as well as the side effects of D and DMD were not affected by the order in which they were administered.

On direct questioning, 37 patients (62%) expressed a preference for D protocol, 14 (23%) preferred DMD, and 9 (15%) experienced no difference. This pattern of preference was not influenced by the order in which the antiemetic protocols were administered. For patients who received D regimen first, 20 patients (67%) preferred D, 6 (20%) favoured the DMD combination, and 4 (13%) found that the two protocols were equal. On the other hand, for patients started on DMD, 17 (57%) favoured D protocol, 8 (27%) preferred DMD, and 5 (16%) could not appreciate any dissimilarity.

**Table IV** Side effects of dexamethasone (D) and dexamethasone, metoclopramide, diphenhydramine (DMD)

| | | *Effect on activity*[a] DMD | | | |
|---|---|---|---|---|---|
| | | *0* | *1* | *2* | *Total* |
| | 0 | 17 | 14 | 9 | 40 |
| | 1 | 6 | 3 | 3 | 12 |
| D | 2 | 1 | 7 | 0 | 8 |
| | Total | 24 | 24 | 12 | 60 |
| | | ($\chi^2_3=11.2$, $P=0.01$) | | | |
| | | *Effect on appetite*[b] DMD | | | |
| | | *0* | *1* | *2* | *Total* |
| | 0 | 15 | 14 | 13 | 42 |
| | 1 | 7 | 3 | 3 | 13 |
| D | 2 | 2 | 1 | 2 | 5 |
| | Total | 24 | 18 | 18 | 60 |
| | | ($\chi^2_3=11.4$, $P=0.01$) | | | |
| | | *Sedative effect*[c] DMD | | | |
| | | *0* | *1* | *2* | *Total* |
| | 0 | 12 | 18 | 8 | 38 |
| | 1 | 8 | 8 | 3 | 19 |
| D | 2 | 0 | 0 | 3 | 3 |
| | Total | 20 | 26 | 14 | 60 |
| | | ($\chi^2_3=14.85$, $P=0.002$) | | | |

[a]Activity: 0 (normal), 1 (mild impairment), 2 (moderate–severe impairment); [b]Appetite: 0 (normal), 1 (mild impairment), 2 (moderate–severe impairment); [c]Sedation: 0 (none), 1 (mild), 2 (severe).

**Table V** The main side effects of dexamethasone (D), and dexamethasone, metoclopramide, and diphenhydramine (DMD)

| *Side effect* | *D* | | *DMD* | | *P* |
|---|---|---|---|---|---|
| | Yes | No | Yes | No | |
| Hallucinations | 0 | 60 | 0 | 60 | NS |
| Chills | 0 | 60 | 2 | 58 | NS |
| Euphoria | 4 | 56 | 2 | 58 | NS |
| Diaphoresis | 2 | 58 | 21 | 39 | <0.01 |
| Insomnia | 10 | 50 | 27 | 33 | <0.01 |
| Headache | 12 | 48 | 32 | 28 | <0.01 |
| Dizziness | 8 | 52 | 27 | 33 | <0.01 |
| Diarrhoea | 5 | 55 | 29 | 31 | <0.01 |

### Discussion

The results of this double-blind, crossover, randomized trial demonstrate clearly again that high-dose dexamethasone (D) is an effective antiemetic agent for patients receiving chemotherapy of high emetogenic potential. Effective antinausea and antivomiting activities of 50% and 57% respectively in this study are virtually identical to the 48% and 58% shown in our previous trial with this drug (Ibrahim *et al.*, 1986). On the other hand, the employment of the antiemetic combination of dexamethasone, metoclopramide and diphenhydramine (DMD) prevented nausea and vomiting in 28% and 43% of subjects. However, the difference between the efficacy of the two regimens was not statistically significant. Furthermore, no significant difference could be demonstrated between the two regimens in relation to the employed emetogenic cytotoxic drugs. However, a trend – though was not significant – in favour of D was noted in patients who received *cis*-platin-containing combinations.

In contrast to our findings, Kris *et al.* (1985) obtained effective antiemesis in 81% of their patients treated with an identical dose schedule of DMD. The discrepancy between these two results can be explained by differences in relevant variables. First, our subjects were younger with a median age of 41 years compared to 55 years in the trial of Kris and co-workers. Thus age may be an important factor as it has been shown that elderly patients are more sensitive to the antiemetic effects of metoclopramide (Meyer *et al.*, 1984). Secondly, the differences in the two trial populations, study designs, chemotherapeutic regimens and criteria for evaluating response also provide additional explanations for the disparate results.

It had been noted previously that high-dose dexamethasone is a safe antiemetic (Cassileth *et al.*, 1984; Ibrahim *et al.*, 1986). Our findings reinforce this important observation. On our D regimen, 38 patients (63%) were free from any sedative effects while 40 (67%), and 42 (70%) did not experience any adverse changes in level of activity and appetite respectively. Furthermore, 27 subjects (45%) did not suffer from any complications during the administration of the drug. DMD caused significantly more frequent side effects than D. Using an identical DMD protocol, Kris *et al.* (1985) reported mild sedation (which occurred in 79% of their subjects) and diarrhoea as the only harmful effects. In contrast, in our present study mild to severe sedation was observed in 67% of the patients. Somnolence induced by antiemetics may be advantageous as it may make the act of vomiting more tolerable (Blandford *et al.*, 1979; Krebs *et al.*, 1985). On the other hand, sedation may not only increase the risk of aspirating vomitus but may also pose unacceptable obstacles for ambulatory patients.

Diarrhoea was also a frequent complication of our DMD regimen for it occurred in 29 patients (48%), 19 of whom did not receive *cis*-platin as part of their anticancer therapy. Though *cis*-platin can cause significant diarrhoea (Gralla *et al.*, 1981; Strum *et al.*, 1982) it cannot be incriminated as the responsible agent for the diarrhoea in the majority of those affected. It is well established also that diarrhoea frequently complicates high-dose i.v. metoclopramide therapy (Strum *et al.*, 1984). Thus, Kris *et al.* (1985) in their series of open label consecutive trials on emesis control, demonstrated a marked fall in the incidence of diarrhoea from 42% with metoclopramide alone to only 5% when this drug was combined with dexamethasone and diphenhydramine (DMD protocol) in the same doses as in our study. In another previously published study on patients receiving *cis*-platin, the addition of dexamethasone to the high-dose metoclopramide was also associated with marked reduction in the incidence of the diarrhoea (6%) as compared with during high-dose metoclopramide plus placebo (21%) (Allan *et al.*, 1984). The marked discrepancy between our data and those quoted from other studies regarding the incidence of diarrhoea when dexamethasone is combined with metoclopramide, is difficult to explain. Difference in the study populations, chemotherapy regimens, and the definition and method of assessment of the degree and frequency of diarrhoea are not adequate explanations for such widely divergent results.

The design of our study did not permit a separate evaluation of the antiemetic properties of diphenhydramine. However, its inclusion in the DMD protocol succeeded in protecting patients against adverse extrapyramidal reactions which are known to occur with high-dose metoclopramide (Gralla *et al.*, 1981; Ibrahim *et al.*, 1986; Strum *et al.*, 1984).

Our experience with the short-course DMD regimen does not support the hypothesis that the use of combination antiemetics with different mode of actions is necessarily safer and more effective than a single-agent with known antiemetic potential. The previously reported efficacy and safety of DMD Kris *et al.* (1985), could not be confirmed in our study. Thus, the short-course advantage of DMD which makes this protocol attractive for use in outpatients is almost nullified by increased adverse effects which would be even more intolerable in an outpatient environment.

Though it has been shown that there is a relative lack of dose response relationship for dexamethasone (Drapkin *et al.*, 1982), the optimal dose of this drug has yet to be determined. Therefore, it is possible that a DMD dose-schedule in which dexamethasone is increased and given orally and more frequently, while the dose of metoclopramide is reduced and that of diphenhydramine is left unaltered, might prove more effective and as safe as high-dose dexamethasone alone. On the other hand, a marked reduction in the metoclopramide dose might have an adverse influence on the antiemetic potency of the combination. A higher dose schedule of all the constituent agents would almost certainly be associated with a greater incidence of unacceptable harmful effects. Therefore, the need for evaluation of other antiemetic regimens which are not only suitable and convenient but also safe for outpatient use is urgent.

The authors acknowledge the expert assistance of Dr Mohamed Naguib.

### References

AAPRO, M.S. & PLEZIA, P.M. (1983). Double-blind, crossover study of antiemetic efficacy of high-dose dexamethasone versus high-dose metoclopramide. *Proc. Am. Soc. Clin. Oncol.*, **2**, 93. (*Abstract*)

ALLAN, S.G., CORNBLEET, M.A., WARRINGTON, P.S., GOLLAND, I.M., LEONARD, R.C.F. & SMYTH, J.F. (1984). Dexamethasone and high dose metoclopramide: Efficacy in controlling *cis*-platin induced nausea and vomiting. *Br. Med. J.*, **289**, 878.

ALLEN, J.C., REILLY, L.K., GRALLA, R.J., TYSON, L.B., CAPARROS, B.I. & CHANG, H.S. (1983). Phase I study of metoclopramide in children. *Proc. Am. Soc. Clin. Oncol.*, **2**, 433. (*Abstract*)

BLANDFORD, R.L., DOWDIE, J.R., STEPHENS, M.R. & WALKER, D.M. (1979). Sicca syndrome associated with idiopathic haemochromatosis. *Br. Med. J.*, **1**, 1323.

BRUERA, E.D., ROCA, E., CEDARO, L., CHACON, R. & ESTEVEZ, R. (1983). Improved control of chemotherapy-induced emesis by the addition of dexamethasone to metoclopramide in patients resistant to metoclopramide. *Cancer Treat. Rep.*, **67**, 381.

CASSILETH, P.A., LUSK, E.J., TORRI, S. & GERSON, S.L. (1984). Antiemetic efficacy of high-dose dexamethasone in induction therapy in acute non-lymphocytic leukemia. *Ann. Int. Med.*, **100**, 701.

CASSILETH, P.A., LUSK, E.J., TORRI, S., DINUBILE, N. & GERSON, S.L. (1983). Antiemetic efficacy of dexamethasone therapy in patients receiving cancer chemotherapy. *Arch. Int. Med.*, **143**, 1347.

DRAPKIN, R.L., SOKOL, G.H., PALADINE, W.J., POLACKWICK, R. & LYMAN, G. (1982). The antiemetic effect and dose response of dexamethasone in patients receiving *cis*-platinum. *Proc. Am. Soc. Clin. Oncol.*, **23**, 61. (*Abstract*)

GRALLA, R.G., ITRI, L.M., PISKO, S.E. & 6 others (1981). Antiemetic efficacy of high-dose metoclopramide: Randomized trials with placebo and prochlorperazine in patients with chemotherapy-induced nausea and vomiting. *N. Engl. J. Med.*, **305**, 905.

IBRAHIM, E.M., AL-IDRISSI, H.Y., IBRAHIM, A.W. & 5 others (1986). Antiemetic efficacy of high-dose dexamethasone: Randomized, double-blind, crossover study with high-dose metoclopramide in patients receiving cancer chemotherapy. *Eur. J. Cancer Clin. Oncol.*, **22**, 283.

KREBS, H.B., MYERS, M.B., WHEELOCK, J.B. & GOPLERUD, D.R. (1985). Combination antiemetic therapy in *cis*-platin-induced nausea and vomiting. *Cancer*, **55**, 2645.

KRIS, M.G., GRALLA, R.J., TYSON, L.B. & 6 others (1985). Improved control of *cis*-platin-induced emesis with high-dose metoclopramide and with combinations of metoclopramide, dexamethasone, and diphenhydramine. *Cancer*, **55**, 527.

KRIS, M.G., TYSON, L.B., GRALLA, R.J., ALLEN, J.C. & REILLY, L.K. (1983). Extra-pyramidal reactions with high-dose metoclopramide. *N. Engl. J. Med.*, **309**, 433.

LASZLO, J. (1983). Nausea and vomiting as major complications of cancer chemotherapy. *Drugs*, **25** (Suppl. 1), 1.

LASZLO, J. & LUCAS, V.S., JR. (1981). Emesis as a critical problem in chemotherapy, Editorial. *N. Engl. J. Med.*, **305**, 948.

MARKMAN, M., SHEIDLER, V., ETTINGER, D.S., QUASKEY, S.A. & MELLITS, E.D. (1984). Antiemetic efficacy of dexamethasone. Randomized double-blind, crossover study with prochlorperazine in patients receiving cancer chemotherapy. *N. Engl. J. Med.*, **311**, 549.

MASON, B.A., DAMBRA, J., GROSSMAN, B. & CATALANO, R.B. (1982). Effect control of *cis*-platin-induced nausea with high dose steroids and droperidol. *Cancer Treat. Rep.*, **66**, 243.

MCNEMAR, A. (1955). *Psychological Statistics*. Wiley: New York.

MEYER, B.R., LEWIN, M., DRAYER, D.E., PASMANTIER, M., LONSKI, L. & REIDENBERG, M.M. (1984). Optimizing metoclopramide control of *cis*-platin-induced emesis. *Ann. Int. Med.*, **100**, 393.

MOERTEL, C.G. & REITEMEIER, R.G. (1969). Controlled clinical studies of orally administered antiemetic drugs. *Gastroenterology*, **57**, 262.

MOERTEL, C.G., REITEMEIER, R.G. & GAGE, R.P. (1963). A controlled clinical evaluation of antiemetic drugs. *JAMA*, **57**, 116.

MORRAN, C., SMITH, D.C., ANDERSON, D.A. & MCARDLE, C.S. (1979). Incidence of nausea and vomiting with cytotoxic chemotherapy: A prospective randomised trial of antiemetics. *Br. Med. J.*, **1**, 1323.

PEROUTKA, S.J. & SNYDER, S.H. (1982). Antiemetics: Neurotransmitter receptor binding predics therapeutic actions. *Lancet*, **i**, 658.

SALLAN, S.E., CRONIN, C., ZELEN, M. & ZINBERG, N.E. (1980). Antiemetic in patients receiving chemotherapy for cancer: A randomized comparison of delta-9-tetrahydrocannabinol and prochlorperazine. *N. Engl. J. Med.*, **302**, 135.

SEIGEL, L.J. & LONG, D.L. (1981). The control of chemotherapy-induced emesis: Review. *Ann. Int. Med.*, **95**, 352.

STRUM, S.B., McDERMED, J.E. & OJELL, R.W. (1982). Intravenous metoclopramide: An effective antiemetic in cancer chemotherapy. *JAMA*, **247**, 2683.

STRUM, S.B., McDERMED, J.E., PILEGGI, J., RIECH, L.P. & WHITAKER, H. (1984). Intravenous metoclopramide: Prevention of chemotherapy induced nausea and vomiting. *Cancer*, **53**, 1432.

WAMPLER, G. (1983). The pharmacology and clinical effectiveness of phenothiazines and related drugs for managing chemotherapy-induced emesis. *Drugs*, **24**(Suppl. 1), 35.